# Comparison of treatment outcome between living donor liver transplantation and sorafenib for patients with hepatocellular carcinoma beyond the Milan criteria

**DOI:** 10.18632/oncotarget.17733

**Published:** 2017-05-10

**Authors:** Yuri Cho, Jeong-Hoon Lee, Dong Hyeon Lee, Eun Ju Cho, Su Jong Yu, Nam-Joon Yi, Kwang-Woong Lee, Yoon Jun Kim, Jung-Hwan Yoon, Kyung-Suk Suh

**Affiliations:** ^1^ Department of Internal Medicine and Liver Research Institute, Seoul National University College of Medicine, Seoul, Republic of Korea; ^2^ Department of Surgery, Seoul National University College of Medicine, Seoul, Republic of Korea; ^3^ Department of Internal Medicine, CHA Gangnam Medical Center, CHA University, Seoul, Republic of Korea; ^4^ Department of Internal Medicine, Seoul Metropolitan Government Seoul National University Boramae Medical Center, Seoul, Republic of Korea

**Keywords:** hepatocellular carcinoma, living donor liver transplantation, sorafenib, MoRAL score, survival

## Abstract

For patients with advanced hepatocellular carcinoma (HCC), sorafenib is the only systemic treatment recommended by international guidelines. We recently reported that HCC patients with a low MoRAL (model to predict tumor recurrence after LDLT) score (≤ 314.8) have excellent treatment outcomes after living-donor liver transplantation (LDLT), even though they are beyond the Milan criteria. In the present study, we investigated whether LDLT offers a better treatment outcome than sorafenib for patients with HCC beyond the Milan criteria according to the MoRAL score. A retrospective cohort study of 325 consecutive patients who were treated with either LDLT (*n* = 122) or sorafenib (*n* = 203) for HCC beyond the Milan criteria from 2005 to 2014 at a tertiary hospital was performed. The primary and secondary endpoints were overall survival (OS) and time-to-progression. When baseline characteristics were balanced using inverse probability weighting, OS was significantly longer in the LDLT group than in the sorafenib group (5-year OS rate, 71.9% vs. 4.9%; HR=0.1; *P* < 0.001). The LDLT group exhibited a significantly lower risk of tumor progression (5-year recurrence rate, 34.7% vs. 96%; HR=0.14; *P* < 0.001) than the sorafenib group. The increase in OS with LDLT was predominantly among patients with a low MoRAL score (5-year OS rate, 81.1% vs. 5.8%; HR=0.06; *P* < 0.001) compared with those with a high MoRAL score (5-year OS rate, 28.3% vs. 4.3%; HR = 0.42; *P* = 0.047). Patients with a low MoRAL score and without extrahepatic metastasis or hepatic vein invasion might be good candidates for LDLT instead of sorafenib treatment if there is a willing living related donor.

## INTRODUCTION

Liver transplantation has been widely accepted as the treatment of choice for both the early stages of hepatocellular carcinoma (HCC) [1] and end-stage liver disease [2]. Recently, there has been a gradual expansion of the liver transplantation recipient criteria due to excellent overall survival (OS). Expanding the selection criteria for patients beyond the Milan criteria has been suggested by some pioneers, resulting in more patients who are beyond the Milan criteria being cured by liver transplantation at the expense of a higher rate of recurrence. Patients with HCC exceeding the Milan criteria have shown a high recurrence rate after liver transplantation - up to 50% at 3 years [3]. However, some of these patients with favorable tumor biology achieve long-term recurrence-free-survival and are probably cured; these patients might be good candidates for living-donor liver transplantation (LDLT) [4–6].

In Asia, LDLT is more frequently performed than deceased-donor liver transplantation (DDLT) due to a shortage of deceased donors and possibly also for cultural and religious reasons [7]. Therefore, guidelines detailing how to select recipient candidates for LDLT in advanced HCC are essential. We previously established and externally validated a model to predict tumor recurrence after LDLT for HCC beyond the Milan criteria: the model to predict tumor recurrence after LDLT (MoRAL) score. The MoRAL score uses serum alpha-fetoprotein (AFP) and protein induced by vitamin K absence-II (PIVKA-II) levels in the formula: 11 × √PIVKA + 2 × √AFP. The score can predict tumor recurrence after LDLT and identifies those patients with the potential for low recurrence and long-term OS. The 5-year OS and the 5-year cumulative tumor recurrence rates are approximately 80% and 30%, respectively, in patients beyond the Milan criteria with a low MoRAL score (≤ 314.8) who might be good candidates for LDLT [8].

For patients with advanced HCC, sorafenib is the only treatment proven to have a survival benefit over the best supportive care. However, the median survival and the time-to-radiologic progression are only 2.3 to 3 months longer for patients treated with sorafenib than for those given placebo. The median OS was 10.7 months in the sorafenib group in the Phase III Study of Sorafenib in Patients With Advanced Hepatocellular Carcinoma (SHARP trial) [9, 10]. No consistent survival benefit has been reported for other anticancer systemic therapeutic agents for HCC in approximately 100 randomized studies that investigated intraarterial systemic chemotherapy (doxorubicin and platinum), various hormonal therapies (tamoxifen and antiandrogens), and immunotherapy (interferon alfa) [11–14]. Although locoregional therapies (e.g., radiofrequency ablation, percutaneous ethanol injection, and transarterial chemoembolization) or radiotherapy (conformal or stereotactic) are recommended for patients with unresectable HCC [15, 16], only a few of these are proven to have a survival benefit greater than 1 year [17, 18]. The patients’ underlying poor liver function often limits the utility of locoregional therapies due to the risk of liver failure after treatment. Therefore, for patients with unresectable HCC, LDLT is considered an alternative treatment option.

To date, there has been no study analyzing the survival benefit of LDLT over sorafenib for patients beyond the Milan criteria. The present study analyzes whether LDLT offers a better treatment outcome than sorafenib for HCC patients beyond the Milan criteria, according to their MoRAL scores.

## RESULTS

### Baseline characteristics

A total of 325 consecutive patients (122 patients in the LDLT group, 203 patients in the sorafenib group) were analyzed. Table 1 shows the baseline characteristics of the two groups. There were significant differences in the baseline age, Child-Pugh score, and American Joint Committee on Cancer (AJCC), 7th edition, T classification between groups.

**Table 1:** Baseline characteristics

Clinical characteristics	LDLT group (*n* = 122)	Sorafenib group (*n* = 203)	*P*-value
Age (years)	59 (35−75)	63 (40−86)	0.01
Male, No. (%)	104 (85.2%)	179 (88.2%)	0.65
Etiology, No. (%)	100/13/5/4	145/32/12/14	0.26
HBV/HCV/alcohol/others	(82.0/10.7/4.1/3.3%)	(71.4/15.8/5.9/6.9%)	
Child-Pugh class, No. (%)	50/45/27	64/134/5	< 0.001
A/B/C	(41.0/36.9/22.1%)	(31.5/66.0/2.5%)	
BCLC stage, No. (%)	60/35/27	108/90/5	< 0.001
B/C/D	(49.2/28.7/22.1%)	(53.2/44.3/2.5%)	
AJCC 7th T classification			< 0.001
T1	0	2 (1.0%)	
T2	25 (22.7%)	64 (31.5%)	
T3a	29 (26.4%)	24 (13.3%)	
T3b	20 (18.2%)	86 (47.8%)	
T4	36 (32.7%)	4 (2.2%)	
MELD score	8.9 (2.5−22.6)	6.9 (1.2−14.5)	< 0.001
AFP (ng/mL)	21.3 (1.3−1708000)	212.3 (1.3−427000)	< 0.001
PIVKA-II (mAU/mL)	57 (6−75000)	308 (2.9−75000)	< 0.001
Maximal tumor size (cm)	2.9 (1.2−13.8)	5 (1−10)	< 0.001
Number of nodules	4 (1−30)	5 (1−28)	0.42
Type of HCC, No. (%)			0.27
Nodular/Diffuse or infiltrative	99/23 (81.1/18.9%)	153/50 (75.4/24.6%)	
Portal vein invasion, No. (%)	47 (38.5%)	80 (39.4%)	0.91
Location of portal vein invasion, No. (%)			0.15
Intrahepatic	28 (59.6%)	37 (46.3%)	
Extrahepatic	19 (40.4%)	43 (53.7%)	
MoRAL score	113.2 (33.7−3928.3)	266.7 (14.1−3969.3)	< 0.001

Data are expressed as n (%) or median with range.

Abbreviation: LDLT, living donor liver transplantation; HBV, hepatitis B virus; HCV, hepatitis C virus; BCLC, Barcelona clinic liver cancer; AJCC, American Joint Committee on Cancer; MELD, Model For End-Stage Liver Disease; AFP, alpha-fetoprotein; PIVKA-II protein induced by vitamin K absence-II; HCC, hepatocellular carcinoma; MoRAL, model to predict tumor recurrence after LDLT.

The median age was 59 years in the LDLT group and 63 years in the sorafenib group (*P* = 0.01). Hepatitis B virus was the most common etiology of HCC in both groups. Only 2.5% of patients in the sorafenib group were Child-Pugh class C, less than the 22.1% in the LDLT group (*P* < 0.001). Forty-seven patients (38.5%) in the LDLT group and 80 patients (39.4%) in the sorafenib group had portal vein invasion, respectively (*P* = 0.91). After inverse probability weighting (IPW), 60 patients (49.2%) in the LDLT group and 80 patients (39.4%) in the sorafenib group had portal vein invasion, respectively (*P* = 0.13).

The median serum AFP and PIVKA-II levels were higher in the sorafenib group than in the LDLT group. The median MoRAL score in the sorafenib group was higher than that of the LDLT group (266.7 *vs*. 113.2; *P* < 0.001). The median wait time prior to LDLT (from HCC diagnosis to LDLT) was 6.9 months (range, 0.3–131.1 months). There was no donor mortality.

### Comparison between LDLT and sorafenib groups for OS and time-to-progression (TTP)

After IPW, the baseline characteristics, including age, AJCC T classification, and Child-Pugh class, became more balanced than before weighting and did not differ significantly between groups (Table 2).

**Table 2:** Checking balance

	LDLT group		Sorafenib group		Standardized Effect Size (unweighted)	*P*-Value (unweighted)	Standardized Effect Size (weighted)	*P*-Value (weighted)
	Mean	SD	Mean	SD				
Age (years)	58.6	7.98	62.68	10.73	−0.432	0.04	−0.202	0.46
Sex						0.21		0.30
Male	0.85	0.4	0.89	0.31	−0.262		0.174	
Female	0.15	0.4	0.11	0.31	0.262		−0.174	
AJCC 7th T classification						< 0.001		0.97
T2	0.08	0.27	0.21	0.41	−0.382		−0.095	
T3a	0.12	0.32	0.13	0.33	−0.023		−0.099	
T3b	0.24	0.43	0.63	0.48	−0.850		0.117	
T4	0.56	0.5	0.02	0.14	1.474		0.053	
Child Pugh class						< 0.001		0.93
A	0.16	0.37	0.37	0.48	−0.495		−0.002	
B	0.52	0.5	0.59	0.49	−0.131		−0.058	
C	0.32	0.47	0.04	0.2	0.772		0.111	

Abbreviation: LDLT, living donor liver transplantation; SD, standard deviation; AJCC, American Joint Committee on Cancer.

**Figure 1:**
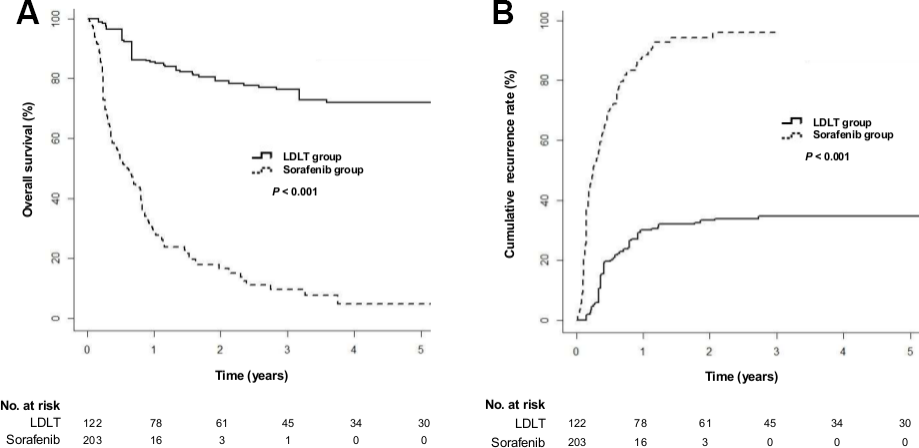
Cumulative overall survival and tumor recurrence rates in the living-donor liver transplantation (LDLT) and sorafenib groups after inverse probability weighting (IPW) (**A**) Cumulative overall survival rates (*P* < 0.001 by log-rank test). (**B**) Cumulative tumor-recurrence rates (*P* < 0.001 by log-rank test).

When the baseline characteristics were balanced using IPW, the LDLT group showed a significantly lower risk of death than the sorafenib group (Figure 1A; hazard ratio [HR] = 0.1; 95% confidence interval [CI], 0.05−0.2; *P* < 0.001). The median OS of the sorafenib group was 8.3 months (interquartile range [IQR], 3.2–18.2 months), while that of the LDLT group was not reached (IQR, 34.1 months–not applicable). The 5-year OS rate was 71.9% in the LDLT group and 4.9% in the sorafenib group. The LDLT group also had a lower risk of tumor progression (Figure 1B; HR = 0.14; 95% CI, 0.08−0.24; *P* < 0.001). The 5-year cumulative tumor recurrence rate was 34.7% in the LDLT group and 96.0% in the sorafenib group.

Multivariate Cox regression analyses for OS (Table 3) and TTP (Table 4) was performed before and after IPW. The independent predictors of OS and TTP included the AJCC T classification and the treatment group (LDLT *vs*. sorafenib). The treatment group was the most significant predictor of both OS and TTP.

**Table 3:** Univariate and multivariate Cox proportional hazard analyses for overall survival

Variable	Univariate analysis		Multivariate analysis	
	HR (95% CI)	*P*-value	HR (95% CI)	*P*-value
Age (years)	1.01 (1.0–1.03)	0.13		
Sex, female (*vs*. male)	0.84 (0.51–1.39)	0.51		
AJCC 7th T classification (*vs*. T1)				
T2	0.13 (0.03–0.52)	0.004	0.14 (0.03–0.60)	0.01
T3a	0.13 (0.03–0.54)	0.005	0.37 (0.09–1.59)	0.18
T3b	0.30 (0.07–1.21)	0.09	0.37 (0.09–1.51)	0.16
T4	0.09 (0.02–0.41)	0.002	0.59 (0.13–2.68)	0.50
Child-Pugh class (*vs*. A)				
B	1.27 (0.90–1.80)	0.17	0.87 (0.61–1.23)	0.43
C	0.45 (0.23–0.86)	0.02	0.99 (0.47–2.11)	0.98
Treatment group (*vs*. sorafenib group)				
LDLT group	0.12 (0.08–0.19)	< 0.001	0.07 (0.04–0.13)	< 0.001
**After IPW**				
Age (years)	0.99 (0.95–1.02)	0.50		
Sex, female (vs. male)	1.15 (0.63–2.07)	0.65		
AJCC 7th T classification (*vs*. T1)				
T2	0.11 (0.03–0.43)	0.002	0.17 (0.04–0.67)	0.01
T3a	0.17 (0.04–0.72)	0.02	0.42 (0.10–1.76)	0.24
T3b	0.20 (0.05–0.79)	0.02	0.44 (0.11–1.76)	0.25
T4	0.22 (0.05–0.99)	0.04	0.51 (0.11–2.31)	0.38
Child-Pugh class (*vs*. A)				
B	0.90 (0.53–1.54)	0.70		
C	0.74 (0.34–1.61)	0.45		
Treatment group (*vs*. sorafenib group)				
LDLT group	0.12 (0.06–0.23)	< 0.001	0.10 (0.05–0.20)	< 0.001

Abbreviation: HR, hazard ratio; CI, confidence interval; AJCC, American Joint Committee on Cancer; LDLT, living donor liver transplantation; IPW, inverse probability weighting

**Table 4:** Univariate and multivariate Cox proportional hazard analyses for time-to-progression

Variable	Univariate analysis		Multivariate analysis	
	HR (95% CI)	*P*-value	HR (95% CI)	*P*-value
Age (years)	1.01 (1.0–1.03)	0.25		
Sex, female (*vs*. male)	0.78 (0.48–1.28)	0.33		
AJCC 7th T classification (*vs*. T1)				
T2	0.59 (0.08–4.24)	0.60		
T3a	0.28 (0.04–2.13)	0.22		
T3b	0.72 (0.10–5.2)	0.74		
T4	0.38 (0.05–2.8)	0.34		
Child-Pugh class (*vs*. A)				
B	1.5 (1.07–2.1)	0.02	1.08 (0.77–1.52)	0.66
C	0.39 (0.19–0.79)	0.01	0.82 (0.39–1.76)	0.62
Treatment group (*vs*. sorafenib group)	0.15 (0.10–0.22)	< 0.001	0.16 (0.10–0.25)	< 0.001
LDLT group				
**After IPW**				
Age (years)	0.99 (0.95–1.02)	0.39		
Sex, female (*vs*. male)	0.94 (0.50–1.76)	0.83		
AJCC 7th T classification (*vs*. T1)				
T2	0.49 (0.35–0.68)	< 0.001	0.94 (0.70–1.28)	0.70
T3a	0.36 (0.21–0.61)	< 0.001	0.99 (0.59–1.69)	1.00
T3b	0.54 (0.34–0.84)	0.01	1.29 (0.86–1.94)	0.22
T4	0.86 (0.48–1.53)	0.61	2.33 (1.29–4.23)	0.01
Child-Pugh class (*vs*. A)				
B	1.19 (0.70–2.05)	0.52		
C	0.64 (0.28–1.46)	0.29		
Treatment group (*vs*. sorafenib group)	0.15 (0.09–0.25)	< 0.001	0.14 (0.08–0.24)	< 0.001
LDLT group				

Abbreviation: HR, hazard ratio; CI, confidence interval; AJCC, American Joint Committee on Cancer; LDLT, living donor liver transplantation; IPW, inverse probability weighting

**Figure 2:**
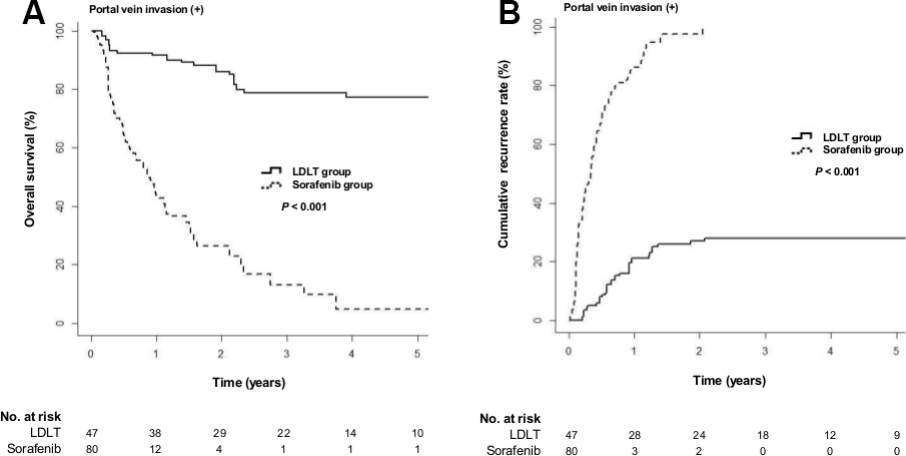
Cumulative overall survival and tumor recurrence rates in the LDLT and sorafenib groups after IPW for the patients with portal vein invasion (**A**) Cumulative overall survival rates (*P* < 0.001 by log-rank test). (**B**) Cumulative tumor recurrence rates (*P* < 0.001 by log-rank test).

When we performed subgroup analysis for the patients with portal vein invasion, the LDLT group still showed a significantly lower risk of death (Figure 2A; HR = 0.11; 95% CI, 0.05−0.27; *P* < 0.001) and a significantly lower risk of tumor recurrence (Figure 2B; HR = 0.2; 95% CI, 0.1−0.39; *P* < 0.001) than the sorafenib group.

### Subgroup analysis according to the MoRAL score for OS and TTP

We performed subgroup analysis by dividing patients into two groups according to the MoRAL score, using a cutoff of 314.8. We then compared the LDLT and sorafenib group for OS and TTP.

**Figure 3:**
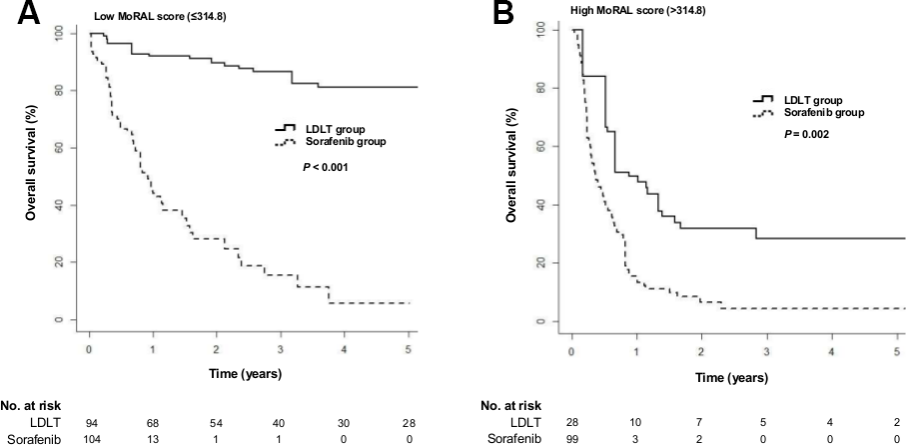
Cumulative overall survival in the LDLT and sorafenib groups according to the MoRAL score using a cut-off of 314.8 (**A**) Cumulative overall survival in patients with a low MoRAL score (≤ 314.8; *P* < 0.001 by log-rank test). (**B**) Cumulative overall survival in patients with a high MoRAL score (> 314.8; *P* = 0.002 by log-rank test)

The increase in OS for LDLT over sorafenib was more predominant in those patients with a low MoRAL score (≤ 314.8) (Figure 3A; HR = 0.06; 95% CI, 0.02−0.17; *P* < 0.001) than in those with a high MoRAL score (> 314.8) (Figure 3B; HR = 0.41; 95% CI, 0.18−0.99; *P* = 0.002). Among the patients with a low MoRAL score, the median OS was not reached in the LDLT group and 13.7 months (IQR, 6.1–28.7 months) in the sorafenib group. The 5-year OS rate was 81.1% in the LDLT group with a low MoRAL score and 5.8% in the sorafenib group with a low MoRAL score. In patients with a high MoRAL score, the median OS was 16.0 months (IQR, 6.6–71.0 months) in the LDLT group and 4.3 months (IQR, 2.8–9.8 months) in the sorafenib group. The 5-year OS rate was 28.3% in the LDLT group with a high MoRAL score and 4.3% in the sorafenib group with a high MoRAL score.

**Figure 4:**
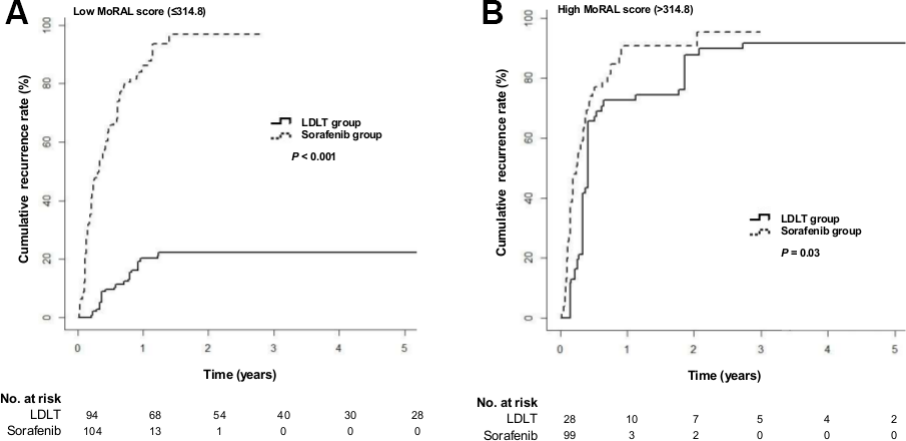
Cumulative tumor recurrence rates in the LDLT and sorafenib groups according to the MoRAL score using a cut-off of 314.8 (**A**) Cumulative tumor recurrence rates in patients with a low MoRAL score (≤ 314.8; *P* < 0.001 by log-rank test) (**B**) Cumulative tumor recurrence rates in patients with a high MoRAL score (> 314.8; *P* = 0.03 by log-rank test).

The increase in TTP for LDLT over sorafenib was also more predominant in those patients with a low MoRAL score (Figure 4A; HR = 0.08; 95% CI, 0.04−0.16; *P* < 0.001) than in those with a high MoRAL score (Figure 4B; HR = 0.53; 95% CI, 0.28−0.91; *P* = 0.03). Among the patients with a low MoRAL score, the median TTP was not reached in the LDLT group and 4.1 months (IQR, 1.6–7.7 months) in the sorafenib group. The 5-year tumor recurrence rate in the LDLT group with a low MoRAL score was only 22.2%, while that in the sorafenib group with a low MoRAL score was 96.8%. In patients with a high MoRAL score, the median TTP was 7.4 months (IQR, 4.0–32.8 months) in the LDLT group and 3.7 months (IQR, 1.4–7.4 months) in the sorafenib group. The 5-year cumulative tumor recurrence rate in the LDLT group with a high MoRAL score was 91.6%, while that in the sorafenib group with a high MoRAL score was 95.4%.

## DISCUSSION

In this study, we found that LDLT confers significantly longer OS and TTP than sorafenib for patients with HCC beyond the Milan criteria. The increase in OS for LDLT over sorafenib is more predominant in those patients with a low MoRAL score (≤ 314.8). Our results suggest that patients beyond the Milan criteria with a low MoRAL score and without extrahepatic metastasis might be good candidates for LDLT rather than sorafenib treatment, if there is a willing living related donor.

Sorafenib is a multitargeted tyrosine kinase inhibitor that inhibits vascular endothelial growth factor receptor, platelet-derived growth factor receptor, b-Raf, Fms-related tyrosine kinase, and c-kit [19]. Sorafenib has limited efficacy and a low objective tumor response rate (partial response rate = 2%, stable disease rate = 40%) [9, 20]. However, sorafenib is still the standard treatment when patients have extrahepatic metastasis or unresectable HCC that is either not suitable for or refractory to transarterial chemoembolization. Approximately half of patients with HCC are candidates for systemic chemotherapy, including sorafenib [21, 22]. The prognosis of those patients is poor, with less than 8 months of OS if untreated. Due to the limited efficacy of sorafenib, there is an urgent need to look beyond this medication and establish a more effective therapy for HCC. According to the Hong Kong Liver Cancer staging criteria, surgical- or locoregional treatment is recommend for the subgroup of patients with advanced HCC, such as multinodular or locally-advanced HCC, if they have preserved liver function [23].

The Milan criteria are considered a universal standard for selecting HCC patients for liver transplantation, limiting the risk of tumor recurrence to an acceptable level [1, 24]. However, the feasibility of liver transplantation for a patient beyond the Milan criteria is still a topic of debate. Although the recurrence rate is high, liver transplantation remains the only possible curative treatment for these patients. Therefore, we analyzed the efficacy of LDLT versus sorafenib for these patients. Liver transplantation provides oncologic and cirrhotic liver clearance. In this regard, liver transplantation differs from other locoregional treatments or systemic chemotherapy. In this study, we found that LDLT might be a successful strategy to prolong OS and TTP for those patients. To our knowledge, this is the first report showing a significant survival benefit for LDLT over sorafenib for patients with HCC beyond the Milan criteria.

Over many years, several groups have suggested criteria to limit liver transplantation to HCC patients with a good prognosis. Currently, patients with HCC beyond the Milan criteria do not have equal access to DDLT as those with HCC within the Milan criteria [25]. In LDLT, the donor frequently wishes to donate, even if the recipient has HCC beyond the Milan criteria. Although LDLT is not a curative treatment, it is palliative and prolongs OS. Therefore, systems to provide the most accurate prognostic evaluation for likely participants become increasingly important. Recently, our group developed and validated a model predicting HCC recurrence after LDLT (the MoRAL score) for those patients beyond the Milan criteria, including those with advanced HCC, based on reproducible predictors including serum AFP and PIVKA-II levels. Patients with a high MoRAL score might have more aggressive tumor biology, leading to a worse outcome. Therefore, we suggested that LDLT should be considered as a treatment option for patients with a low MoRAL score [8].

In the current study's subgroup analyses according to MoRAL score, we found that the gain in OS and TTP for LDLT over sorafenib is more predominant in those patients with a low MoRAL score (81.1% *vs*. 5.8% for 5-year OS rate, 22.2% *vs*. 96.8% for 5-year tumor recurrence rate), compared with those with a high MoRAL score (28.3% *vs*. 4.3% for 5-year OS rate, 91.6% *vs*. 95.4% months for 5-year tumor recurrence rate). Tumor recurrence after liver transplantation has a poor prognosis because immunosuppression is a well-known risk factor for tumor growth [26]. Patients with a high MoRAL score have aggressive tumor biology, leading to a high tumor-recurrence rate. Therefore, the gain in TTP for a patient with a high MoRAL score is lower than that for a patient with a low MoRAL score, leading to a smaller gain in OS. However, it is worth noting that among those patients with a high MoRAL score, LDLT is still superior to sorafenib in OS and TTP. For a patient with a high MoRAL score, the gain in OS and TTP is lower than that seen in a patient with a low MoRAL score, but it is still better than sorafenib, the current standard treatment. Therefore, LDLT might be considered a treatment option for patients with a high MoRAL score.

In the multivariate analysis, AJCC T classification did not stand out as a prognostic factor for OS and TTP. The LDLT group had more advanced intrahepatic tumors (higher T classification) than the sorafenib group at baseline and AJCC T classification was a confounding factor for the treatment group (*P* < 0.001). Actually, there was a significant association between AJCC T classification and OS in univariate analysis. If we omit treatment group from multivariate analysis, T classification was an independent prognostic factor for both OS and TTP (data not shown). It seems that T classification failed to become an independent prognostic factor in the multivariate analysis (both before and after IPW), probably because treatment group (LDLT *vs*. sorafenib) was the strongest independent prognostic factor and there was a confounding effect between treatment group and T classification.

Our study has several limitations. First, this study was performed in Korea, where 70% to 80% of HCC patients are infected with hepatitis B virus [27]. Therefore, our results might not be generalizable, since most Western countries have HCC patients with different underlying etiologies such as hepatitis C virus infection. Second, there might be an ethical issue in performing liver transplantation in beyond the Milan criteria patients. However, we followed ethical principles, including autonomy and nonmaleficence [28]. Third, this was a retrospective cohort study with inherent limitations including selection bias; we therefore performed IPW to minimize bias. However, some unmeasured difference may not be balanced. Further prospective studies are necessary to confirm superior treatment outcome of LDLT over sorafenib.

In conclusion, for HCC patients beyond the Milan criteria, LDLT demonstrates significantly longer OS and TTP than sorafenib. Therefore, patients beyond the Milan criteria with a low MoRAL score and without extrahepatic metastasis might be good candidates for LDLT rather than sorafenib treatment, if there is a willing living related donor.

## MATERIALS AND METHODS

### Study subjects

We retrospectively analyzed consecutive patients who were treated with either LDLT or sorafenib between June 2005 and December 2014 at Seoul National University Hospital, Seoul, Korea. The diagnosis of HCC was based on the noninvasive criteria of the American Association for the Study of Liver Diseases or on the histologic diagnosis [29]. All donors received psychiatric counseling to determine whether they were suitably motivated to undergo LDLT. In both LDLT and sorafenib groups, patients were with HCC which was unresectable and contraindicated or refractory to transarterial chemoembolization. In patients with HCC beyond the Milan criteria, LDLT was performed only for those who were willing to undergo LDLT. None of the patients in the LDLT group were treated with sorafenib prior to liver transplantation. Patients with extrahepatic metastasis and hepatic vein invasion were excluded. In the sorafenib group, patients received oral sorafenib 400 mg twice daily. Treatment with sorafenib continued until either unacceptable toxicity or disease progression occurred.

Pretreatment staging included dynamic liver computed tomography (CT), magnetic resonance imaging, positron emission tomography-CT, bone scanning, and low-dose chest CT. The demographic findings included patient sex, age, viral status, pretransplant AFP and PIVKA-II levels, Child-Pugh class, tumor size, and number of tumors according to pretransplant imaging studies; the presence of portal vein invasion was also noted.

The safety of the living donor was of primary concern. All LDLTs were performed after obtaining fully informed written consent and multidisciplinary approval including internal medicine, surgery, psychiatry, and radiology. The study protocol conformed to the ethical guidelines of the World Medical Association Declaration of Helsinki and was approved by the institutional review board of Seoul National University Hospital (IRB No. 1305-561-490).

### Assessment

Data on clinical and laboratory findings were collected retrospectively from all patients by reviewing their electronic medical records. One radiologist independently reviewed all radiologic images, blinded to survival data, to determine the number and size of tumors, presence or absence of vascular invasion, treatment response, and recurrence. In the LDLT group, patients underwent dynamic CT or magnetic resonance imaging every 2 to 4 months for the first 2 years after liver transplantation and every 3 to 6 months thereafter. In the sorafenib group, patients underwent dynamic CT or magnetic resonance imaging every 4 to 6 weeks during treatment, and adverse events were assessed during the first 3 to 4 weeks of therapy. Patients who discontinued sorafenib treatment due to severe adverse events were not included in the analysis.

The primary endpoint was OS, defined as the time from the date of diagnosis until the date of death from any cause. The secondary endpoints included TTP.

### Statistical analysis

The baseline patient characteristics were expressed as the median (range). To minimize selection bias and better describe the treatment effect of different modalities, baseline characteristics were balanced using IPW. Propensity scores were calculated using a logistic regression model. We predicted the probability for each patient on the basis of the pretreatment variables. After the inverse probabilities of the propensity score weight was generated, the 2 groups were balanced using IPW.

The Kaplan-Meier method was used to estimate OS and TTP. Multivariate Cox proportional hazards regression analysis was performed to compute the HR. Patients were divided into two groups according to the MoRAL score using a cutoff of 314.8. MoRAL-score subgroup analysis was then performed to compare the LDLT and sorafenib groups.

Statistical analyses were performed using PASW STATISTICS 22.0 (SPSS Inc., Chicago, IL, USA) and R language, version 3.01 (R Foundation for Statistical Computing, Vienna, Austria). All statistical tests were 2-sided and conducted in an explorative manner with a significance level of 0.05.

## Abbreviations

HCC: hepatocellular carcinoma
LDLT: living donor liver transplantation
DDLT: deceased-donor liver transplantation
MoRAL: model to predict tumor recurrence after LDLT
AFP: alpha-fetoprotein
PIVKA-II: protein induced by vitamin K absence-II
OS: overall survival
TTP: time-to-progression
CT: computed tomography
HR: hazard ratio
CI: confidence interval
IPW: inverse probability weighting
AJCC: American Joint Committee on Cancer
BCLC: Barcelona clinic liver cancer
MELD: model for end-stage liver disease
IQR: interquartile range.
